# FACTORS RELATED TO GENERATIVITY OF OLDER READING ALOUD VOLUNTEERS DURING THE COVID-19 PANDEMIC: REPRINTS STUDY

**DOI:** 10.1093/geroni/igad104.2664

**Published:** 2023-12-21

**Authors:** Tomoya Takahashi, Kyoko Fujihira, Tomoya Sagara, Koji Fujita, Hiroyuki Suzuki, Hiroko Matsunaga, Hiroshi Murayama, Yoshinori Fujiwara

**Affiliations:** Tokyo Metropolitan Institute for Geriatrics and Gerontology, Tokyo, Tokyo, Japan; Tokyo Metropolitan Institute of Geriatrics and Gerontology, Tokyo, Tokyo, Japan; Tokyo Metropolitan Institute for Geriatrics and Gerontology, Tokyo, Tokyo, Japan; Tokyo Metropolitan Institute for Geriatrics and Gerontology, Tokyo, Tokyo, Japan; Tokyo Metropolitan Institute of Geriatrics and Gerontology, Tokyo, Tokyo, Japan; Tokyo Metropolitan Institute of Gerontology, Tokyo, Tokyo, Japan; Tokyo Metropolitan Institute of Gerontology, Tokyo, Tokyo, Japan; Tokyo Metropolitan Institute for Geriatrics and Gerontology, Tokyo, Tokyo, Japan

## Abstract

Volunteer activities was hampered by the COVID-19 pandemic. This study focused on generativity of older volunteers’ activities during the COVID-19 pandemic and examined the factors associated with one. A self-administered questionnaire survey was conducted among 453 older adults belonging to REPRINTS, a volunteer picture book reading organization from October 2021 to January 2022. 404 respondents were obtained, and 277 respondents (mean age: 73.4, SD: 6.1, female: 91.2%) with no missing responses were included in the analysis. Analysis items were Revised Japanese Version of Generativity Scale (JGS-R) composed of 3 factors (generational interest, behavior and achievement), satisfaction and burden of activities, frequency of activities in the last month, Child Image Scale for Older Adults composed of 5 factors (protection, purity, self-centeredness, creativity and self-reliance), a shortened version of the Help Seeking Scale, physical and mental health (subjective health, TMIG-13, J-WHO-5), living conditions, gender, and age. Multiple regression analysis was used with each subscale score in JGS-R as the dependent variable and others as independent variables. The results showed there was a significant association between generational interest and image of creativity, desire for help, mental health, and social roles; between generational behavior and image of purity (-), creativity image, desire for help, resistance to help (-), and social roles; between generational achievement and desire for help, social roles, and gender (male), respectively. In conclusion, physical and mental health, image of the child, and desire for help were associated with older volunteers’ generativity even during the COVID-19 pandemic even during the COVID-19 Pandemic.

